# Glimpses into evolutionary trajectories of SARS-CoV-2: emerging variants and potential immune evasion routes

**DOI:** 10.2217/fmb-2020-0300

**Published:** 2021-05-07

**Authors:** Cleo Anastassopoulou, Yiannis Manoussopoulos, Vicky Lampropoulou, Athanasios Tsakris

**Affiliations:** ^1^Department of Microbiology, Medical School, National & Kapodistrian University of Athens, Athens, Greece; ^2^Laboratory of Virology, Plant Protection Division of Patras, ELGO-Demeter, Patras, Greece; ^3^Laboratory of Immunobiology, Center for Clinical, Experimental Surgery & Translational Research, Biomedical Research Foundation of The Academy of Athens, Athens, Greece

**Keywords:** adaptation, APOBEC, coronavirus, COVID-19, genetic diversity, genome evolution, immune escape, SARS-CoV-2 variants of concern, spike, vaccines

## Abstract

An opinion on the coronaviruses' evolution paradoxes, the continuing adaptation of the SARS-CoV-2 in humans following the zoonotic transmission, and clues into escape routes from host immune responses.

## The paradoxes in the evolution of coronaviruses

The genomes of most RNA viruses are confined to average sizes of 10 kb, partly because of their high mutation rates that, in the absence of proofreading mechanisms, may result in the accumulation of deleterious mutations that would inactivate the virus (error–catastrophe hypothesis) [1,2]. In this low complexity ‘Eigen trap’ with few RNA-protein (RNP) elements, the diversity generated by the typical errors of 1–2 mutations per nucleotide site per replication round by the RNA dependent RNA polymerases during viral RNA synthesis provides the virus population the capacity to adapt to diverse ecological niches and conditions, such as the dynamic environment of different anatomic sites in infected individuals and the hosts’ immune responses [3]. Accordingly, even modestly increased replication fidelity has been shown to lead to reduced viral fitness *in vitro* and pathogenicity in animal models, although low fidelity RNA viruses with higher mutation rates, may also display compromised fitness *in vivo* [4]. Hence, a finely balanced mixture of genetic diversity, on one hand, on which selection can act, possibly actuating episodic evolution affecting only specific domains at times as required for adaptation, and genome stability, on the other, to ensure that structural constraints are not violated, seems to be required for survival and is thus selected through evolution [4].

Up until the characterization of the 41.1 kb genome of planarian secretory cell nidovirus [5], roni- and coronaviruses were considered to be at the upper end of the RNA virus genome size range (26.3–31.7 kb) and complexity among positive-sense ssRNA viruses. The acquisition of a 3’–5’ proofreading exoribonuclease (nsp14-ExoN) that was implicated in controlling RNA replication fidelity and avoidance of the accumulation of excessive numbers of deleterious mutations that would lead to a dramatic loss in fitness, is thought to have contributed to genome expansion. Thus, the ancestor of these large nidoviruses arose, exemplified by the 20.2 kb genome of the first insect (mosquito)-borne nidovirus, Nam Dinh virus [3]. The discoveries of Nam Dinh virus and planarian secretory cell nidovirus provided the missing evolutionary links in the transition from small to large nidoviruses, concomitantly starting to bridge, in essence, the daunting gap for the proposed evolution of contemporary DNA–RNP-based life from primordial RNP entities as primitive life radiated and dominated the earth.

Despite their large and complex genomes, the high plasticity and recombination-driven adaptive capacity of coronaviruses is strong as shown by the frequent host-switching events through the exploitation of different cellular receptors by the spike protein [6–8]; and by the emergence, presumably from bats, late in 2019 in Hubei Province, China, and rapid spread among the human population worldwide, of SARS-CoV-2 [9].

## The adaptation of SARS-CoV-2 in humans following the zoonotic transmission

The pandemic SARS-CoV-2 that causes COVID-19, has claimed more than 2.9 million lives globally. Although geographically defined clusters that recapitulated the early routes of international spread were described, the remarkably low virus diversity recorded in the early phases of the pandemic (with a midrange substitution rate of 3 × 10^-4^ substitutions/site/year), complicated epidemiological analyses [10]. C→U transitions comprised about half of sequence changes, with an eightfold base frequency normalized directional asymmetry between C→U and U→C substitutions. Of note, similarly elevated ratios were also observed in the other epidemic coronaviruses that emerged recently in evolutionary time (SARS-CoV-1 in 2002 and MERS-CoV in 2012), while decreasing ratios were found in the four endemic human coronaviruses (HCoV-NL63, -OC43, -229E and -HKU1) [10].

Contrary to initial expectations of a slow evolutionary pace of SARS-CoV-2, a number of genomic alterations have been recorded in recent months, as the adaptation of this novel coronavirus in humans continues. The first such change, the D614G amino acid substitution in the spike protein, which prevailed globally soon after its surfacing in February 2020 [11], was shown to be associated with increased viral transmission through a shifted conformation toward a favored human angiotensin-converting enzyme 2 binding-competent state; however, this gain in infectivity likely came at a cost to the neutralization properties of the virus [12,13]. Additional ‘variants of concern’ (VOC), due to their suggested impact on transmission and virulence, are continuously recorded [14].

In September 2020, a lineage that arose in the UK was found to harbor a constellation of 23 genomic mutations, including some amino acid-altering mutations in the spike protein [14]. Increased transmissibility arguably characterizes this designated as ‘B.1.1.7’ (20I/501Y.V1/B.1.1.7) lineage, based on the rapid displacement of previously circulating viruses in the region where it first appeared in southeast England, and its consequent association with an increased *R*_e_ and elevated viral RNA levels in nasopharyngeal washes, as measured by PCR or RNA sequencing [14]. However, discerning between the action of positive selection and the momentum of the founder effect of a virus already exceedingly transmissible among humans, is very difficult. B.1.1.7 variants are certainly fit or reproductively successful, but they are not necessarily more transmissible biologically.

Additional variants that share some of the B.1.1.7 mutations, have been detected in Brazil (P.1/20J/501Y.V3/B.1.1.248), South Africa (20H/501Y.V2/B.1.351), California (20C/S:452R;/B.1.429) and many other locations around the world [14]. Experiments measuring infectious virus in animal models, or humans for that matter, which would provide solid proof of increased transmissibility, have not been conducted yet for any of these variants. Their potential functional effect on disease severity also remains uncertain at present. According to a recent report by the New and Emerging Respiratory Virus Threats Advisory Group, VOC B.1.1.7 is associated at 40–50% confidence with an increased risk of death compared with non-VOC [15]. Genomic surveillance efforts should be intensified globally to monitor for the emergence of variants and the characterization of their biological properties, especially pertaining to immune evasion, as vaccine rollout and mass vaccination of the world’s population continues at a, perhaps unavoidably, slow rate.

## The selection pressure to escape from host immune responses

Host immune responses after natural infection or immunization, can exert selection pressures on the virus that will likely result in the further exploitation of sequence space in search of escape routes. The correlates of immunity to – and protection from – SARS-CoV-2 are not fully understood yet. However, following the impressive interim efficacy results of candidate vaccines in late phase clinical trials as well as the preliminary real-world vaccination results particularly from Israel, people around the world are anxiously anticipating their distribution, asking emphatically whether they will suffice to end this pandemic. Thus, one of the most pressing questions at present is: will the virus mutate to escape the selection pressure from host immune responses, and, moreover, are any SARS-CoV-2 variants already resistant to licensed vaccines?

Pseudotyped viruses bearing the SARS-CoV-2 spike glycoprotein from the B.1.1.7 lineage were efficiently neutralized by sera from recipients of Moderna’s (mRNA-1273) vaccine; in contrast, approximately 6.4-fold reduced neutralization titers were found against the South African B.1.351 lineage, prompting Moderna to announce the advancement of a modified vaccine (mRNA-1273.351) to prevent severe COVID-19 from this lineage, too [16]. Neutralization of sera from recipients of the Pfizer/BioNTech’s (BNT162b2) mRNA vaccine to viruses engineered with selected changes from both lineages (deletion of amino acids 69/70, N501Y and D614G for B.1.1.7 and E484K + N501Y + D614G for B.1.351, respectively) was not found to be compromised [17]. Johnson & Johnson’s just authorized single shot Ad26.COV2.S COVID-19 vaccine appears to be effective against the B.1.351 variant [18], while AZD1222, the other adenovirus-based vaccine by Oxford–AstraZeneca, does not work well against the South African variant [19].

In a recent study, Weisblum *et al.* [20] provide clues to potential immune evasion routes using a recombinant chimeric VSV/SARS-CoV-2 reporter virus system. With this system, escape mutants from antibody neutralization, which is thought to be key for the protection of the population, can be rapidly generated and assessed. Functional SARS-CoV-2 spike protein variants with resistance-conferring mutations to monoclonal antibodies or convalescent plasma were thus shown to be readily selected *in vitro* [20]. The resistance mutations mapped to the receptor binding domain and N-terminal domain. Importantly, but perhaps not surprisingly, escape mutants to commonly elicited neutralizing antibodies can already be detected at low frequencies in circulating SARS-CoV-2 populations [20].

A granular view of potential antibody escape pathways is presented by a recently released preprint that emphasizes that individual variation should be anticipated in antibody-mediated virus evolution [21]. To define the profile of antibody escape to the SARS-CoV-2 spike protein using COVID-19 convalescent plasma, an approach that comprehensively addresses the effect of all possible mutations on binding to a protein of interest, phage-deep mutational scanning (DMS), was used. The fusion peptide and linker region, upstream of the heptad repeat region 2, were the two regions where antibody binding was common, although escape mutations varied within these immunodominant regions [21]. Individual variation was also evident in less commonly targeted epitopes.

The large proportion of sequence change in SARS-CoV-2 found to comprise C→U hypermutation points to the direction of RNA-editing processes acting within the infected cell as the most plausible explanation. Mourier *et al.* [22] recently reviewed three such human defense mechanisms and their potential implications on SARS-CoV-2 evolution: APOBEC, ROS and ADAR.

APOBEC, typically considered an antiviral mechanism against retroviruses, may also mediate antiviral functions against RNA viruses, since it catalyzes cytosine deamination to uracil in foreign ssDNA and RNA [22]. Extensive C-to-U mutations, the genomic context of which was enriched for APOBEC target sites [23], have been observed in SARS-CoV-2 since the early phases of the pandemic [24,25]. Interestingly, only viruses regularly infecting tissues with high expression of APOBEC and other antiviral proteins exhibited CpG-depletion and U-rich genomes [26]. The study by Simmonds published in late June reported that about half of the observed nonsynonymous mutations in SARS-CoV-2 were the result of C-to-U changes [10], while, according to Mourier *et al.* [22], that frequency was 36.9% as of 2 October 2020, by comparing approximately 80,000 assembled consensus genomes to the SARS-CoV-2 reference genome (MN908947.1). Of note, C→U transitions occurred preferentially in both 5’ U/A and 3’ U/A flanking sequence contexts that are comparable to favored motifs of human APOBEC3 proteins [10]. The evolutionary trajectory of SARS-CoV-2 may be severely restricted as a consequence of the potential depletion of alanine, histidine, glutamine, proline and threonine codons due to the progressive loss of genomic cytosines [27].

Another line of host defenses against viral infections involve ROS that may lead to virus mutagenesis and inactivation through the oxidation of proteins, lipids and nucleic acids [28]. In particular, guanine may be oxidized by ROS to 7,8-dihydro-8-oxo-2’-deoxyguanine (oxoguanine) that can readily base pair with adenine, leading to G-to-T transversions [29]. G-to-U, as well as C-to-A changes, have been hypothesized to be associated with the mutagenic activity of ROS [30].

A-to-G changes may stem from the deamination by ADAR of adenine to inosine (I) that pairs with cytosine. A chief controller of cytoplasmic innate immunity, *ADAR1*, the first of the three human *ADAR* genes, targets dsRNA which can arise during the replication-transcription process of positive-sense ssRNA viruses, including SARS-CoV-2 [31]. Its two isoforms exhibit different expression patterns: ADAR1p110 is constitutively expressed in most tissues, while ADAR1p150 is localized in the nucleus and released to operate in the cytoplasm upon stimulation by interferon [32]. *ADAR1* is also an important regulator of self-tolerance, since unedited dsRNA is interpreted as nonself, leading to the activation of the innate immune sensing response via MDA5 [33].

Both ADAR and APOBEC were found as frequent interactors with SARS-CoV-2 RNA by Schmidt *et al.* [34], who used RNA antisense purification and mass spectrometry in their study in infected human cells. Sequence analyses also showed a role for ADAR-mediated editing of the viral genome [23,35], with a note of caution that detected variation could be due to artifacts introduced during sequencing [35]. Indeed, a few other studies found no evidence of ADAR1 activity acting on SARS-CoV-2 [36,37]. However, the mutations created by ADAR1 do not match those observed in SARS-CoV-2 or other coronaviruses; in fact, the excess of C→U transitions contradicts those induced by ADAR1 [10].

The fact that a host APOBEC-like editing process appears to be driving much of sequence change in SARS-CoV-2 has profound implications for its short- and long-term evolution. Prolonged C→U hypermutation could lead to low G+C contents and base asymmetries in the long run, as observed in bat-derived and endemic human coronaviruses [10]. Will the mutational journey of SARS-CoV-2 that started in a hostile human cellular environment lead to a tolerated symbiosis and, if so, how soon could that be in evolutionary time? For now, the novel coronavirus represents an intriguing paradigm with respect to its diversification in sequence space that stems predominantly from biased, convergent, and context-dependent mutations in addition to neutral changes, rendering the tracking of its evolutionary trajectory and description of its fitness landscape challenging for molecular epidemiology investigations.
